# Microfluidic radiosynthesis of [^18^F]FEMPT, a high affinity PET radiotracer for imaging serotonin receptors

**DOI:** 10.3762/bjoc.13.285

**Published:** 2017-12-29

**Authors:** Thomas Lee Collier, Steven H Liang, J John Mann, Neil Vasdev, J S Dileep Kumar

**Affiliations:** 1 Division of Nuclear Medicine and Molecular Imaging, Massachusetts General Hospital & Harvard Medical School, Boston, MA, USA; 2 Advion, Inc., Ithaca, NY, USA; 3 Molecular Imaging and Neuropathology Division, New York State Psychiatric Institute, New York, NY, USA

**Keywords:** agonist, fluorine-18, 5-HT_1A_, microfluidics, PET

## Abstract

Continuous-flow microfluidics has shown increased applications in radiochemistry over the last decade, particularly for both pre-clinical and clinical production of fluorine-18 labeled radiotracers. The main advantages of microfluidics are the reduction in reaction times and consumption of reagents that often result in increased radiochemical yields and rapid optimization of reaction parameters for ^18^F-labeling. In this paper, we report on the two-step microfluidic radiosynthesis of the high affinity partial agonist of the serotonin 1A receptor, [^18^F]FEMPT (p*K*_i_ = 9. 79; *K*_i_ = 0.16 nM) by microfluidic radiochemistry. [^18^F]FEMPT was obtained in ≈7% isolated radiochemical yield and in >98% radiochemical and chemical purity. The molar activity of the final product was determined to be >148 GBq/µmol (>4 Ci/µmol).

## Introduction

**Figure 1:**
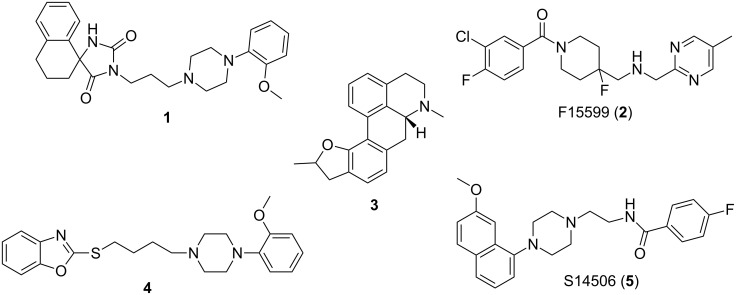
Representative examples of recent 5-HT_1A_R agonists [3–9].

The development of serotonin 1A receptor (5-HT_1A_R) agonist radiotracers for applications in molecular imaging with positron emission tomography (PET) has been avidly sought over the past two decades, albeit with limited success. The current status of serotonin-targeting radiopharmaceuticals was recently reviewed by Paterson et al. [1] and their conclusion was that “the development of PET and single-photon emission computed tomography (SPECT) radioligands to image serotonergic targets is of high interest, and successful evaluation in humans is leading to invaluable insight into normal and abnormal brain function”. A further review by us focusing on 5-HT_1A_R overviewed a number of PET and SPECT tracers that have been tested in vivo with varying efficacy [2]. A few representative compounds which show the structures that have been tested as radiotracers are shown in Figure 1.

The 5-HT_1A_R is implicated in the pathophysiology of a variety of neuropsychiatric and neurodegenerative disease states as well as in the mechanism of action of antidepressants. The 5-HT_1A_R exists in low and high agonist affinity states. The antagonist ligands have similar affinity to the low affinity (LA) and high affinity (HA) conformations of 5-HT_1A_R. However, agonist ligands prefer binding to the HA state of the receptor. This is coupled to G-protein and thus agonist binding gives a more meaningful functional measure of the 5-HT_1A_R that can reflect desensitization and supersensitivity. Significant research has been directed at the differences between agonist and antagonist binding to 5-HT_1A_ receptors in Alzheimer's disease [10] and this interest has led to the development of a high-resolution in vivo atlas for four of the human brain's serotonin receptors and transporters [11].

Antagonist 5-HT_1A_R PET tracers can detect the total receptor binding, but not modifications in the high affinity 5-HT_1A_R binding in disease states or in the context of treatment functionally larger and earlier effects of antidepressants. The development of 5-HT_1A_R agonist PET tracers for the past 2 decades has met with limited success. An arylpiperazine derivative of 3,5-dioxo-(2*H*,4*H*)-1,2,4-triazine radiolabeled with carbon-11 (*t*_½_ = 20.4 min), [^11^C]MPT, is the first successful agonist PET tracer reported for 5-HT_1A_R in non-human primates. The binding of [^11^C]MPT in baboon brain was in excellent agreement with the known distribution of the 5-HT_1A_R. Despite the excellent binding profile of [^11^C]MPT, the slow washout in baboons limits this radiotracer from advancing to human studies. Our recent efforts have focused on the development of fluorine-18 (*t*_½_ = 109.7 min) labelled MPT derivatives, as the longer half-life enables imaging protocols that can provide a better match of the pharmacokinetics of binding to the half-life of the radionuclide, as well as simplified radiochemistry protocols and the long-term goal of distribution for multicenter clinical trials.

Several reports on the use of continuous flow microfluidics for radiofluorination have shown higher yields, with less amount of reagents and shorter reaction times compared to traditional vessel-based techniques [12]. Microfluidic techniques also allow for cost-effective and rapid optimization of reaction parameters for new radiotracers as simultaneous reactions can be carried out. We have recently shown that continuous flow microfluidics is suitable for ^18^F-radiopharmaceutical production studies [13] and have applied this technique in human PET imaging studies [14]. Herein we present the microfluidic synthesis and evaluation of [^18^F]FEMPT as an agonist PET ligand for 5-HT_1A_R.

## Results and Discussion

### Synthesis and binding affinity of FEMPT

**Scheme 1:**
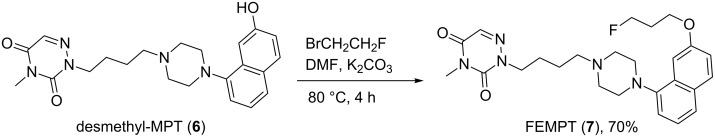
Synthesis of FEMPT (**7**).

Desmethyl-MPT, the radiolabeling precursor, was synthesized as described previously [15]. The reference standard FEMPT (**7**) was synthesized in 70% by reacting desmethyl-MPT (**6**) with 1-bromo-2-fluoroethane in the presence of K_2_CO_3_ (Scheme 1 and Supporting Information File 1).

The in vitro binding assays to establish the potency and selectivity of FEMPT towards 5-HT_1A_R and various other biogenic amines, brain receptors, and transporters were evaluated by the National Institute of Mental Health Psychoactive Drug Screening Program (NIMH-PDSP). FEMPT shows 0.2 nM binding affinity (*K*_i_) to 5-HT_1A_R. The next closest bindings for MPT are Sigma_2_ PC12, H1, 5-HT_7_, and 5-HT_1B_ (Table 1) and are >50 times higher than 5-HT_1A_R. The *K*_i_ values for several other brain receptors and transporters were low (0.1 to 10 μM). Agonist properties of FEMPT on 5-HT_1A_R were estimated by [^35^S]GTPγS binding in membranes of CHO cells which stably express human 5-HT_1A_R. A dose-dependent increase in [^35^S]GTPγS binding was induced by FEMPT. Maximal FEMPT stimulated [^35^S]GTPγS binding *E*_max_ was 100% of that seen with 5-HT and an EC_50_ of 85 nM.

**Table 1:** *K*_i_s of FEMPT for receptors and transporters.

Targets	*K*_i_ values (nM)	Targets	*K*_i_ values (nM)	Targets	*K*_i_ values (nM)

5-HT_1A_	0.2	adrenergic_α1_	180	D_1_	>10,000
5-HT_1B_	122.5	adrenergic_αB_	196	D_2_	80
5-HT_2A_	406	adrenergic_αD_	142	D_3_	35
5-HT_2B_	12	adrenergic_α2A_	346	D_4_	24
5-HT_2C_	343	adrenergic_α2B_	403	D_5_	>10,000
5-HT_3_	>10,000	adrenergic_α2C_	400	DAT	407.4
5-HT_5A_	2340	adrenergic_β1_	1300	sigma_2_ PC12	10
5-HT_6_	71	adrenergic_β2_	202	DAT	407.4
5-HT_7_	11	adrenergic_β3_	564	DOR	>10,000
A_2_, A_3_, A_4_	>10,000	H_1_	11	EP	>10,000
BZP	>10,000	H_2_	1364	GABA	>10,000
Ca^2+^	>10,000	H_3_, H_4_	>10,000	smoothened	>10,000
AMPA	>10,000	hERG	>10,000	Y_2_	>10,000
NET	6980	KOR	1423	SERT	6144
NK	>10,000	M	>10,000	sigma_2_	>10,000
sigma_1_	1014	MDR1	>10,000	VMAT_1,2_	>10,000
V_1_, V_2_	>10,000	MOR	>10,000	NT_1_	>10,000
Na^+^ channel	>10,000	mGluR	>10,000	imidazoline	>10,000
CB_1_, CB_2_	>10,000	NMDA	>10,000		
5-HT_1A_R *E*_max_	100%	EC_50_	85 nM		

### Microfluidic chemistry

**Scheme 2:**
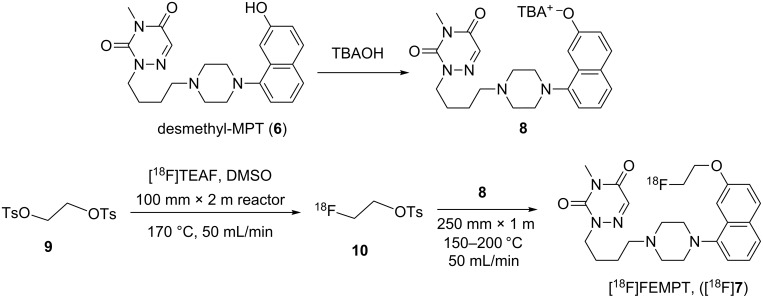
Radiosynthetic scheme for the microfluidic flow synthesis of [^18^F]fluoroethyltosylate (**10**) and [^18^F]FEMPT (**7**).

Reaction optimization of radiofluorination methods vary depending on the microfluidic systems being used but we can typically optimize ^18^F-labeling reaction conditions in one or two days from a single batch of [^18^F]fluoride. This is significantly more efficient than classical vial-based radiofluorination methods which, generally involves several experimental days and analysis as each reaction, including azeotropic drying of [^18^F]fluoride, must be carried out individually. The microfluidic radiosynthesis of [^18^F]FEMPT was optimized by treating the reactions as 2 individual steps, using the Discovery mode of the Advion NanoTek^®^ microfluidic synthesizer [16]. The first step is the preparation of the labeling reagent [^18^F]fluoroethyltosylate (**10**) via tetraethylammonium fluoride ([^18^F]TEAF). The second step is the reaction of the [^18^F]fluoroethyltosylate (**10**), with the FEMPT precursor **8** (Scheme 2).

#### Step 1: Synthesis of [^18^F]fluoroethyltosylate (**10**)

**Figure 2:**
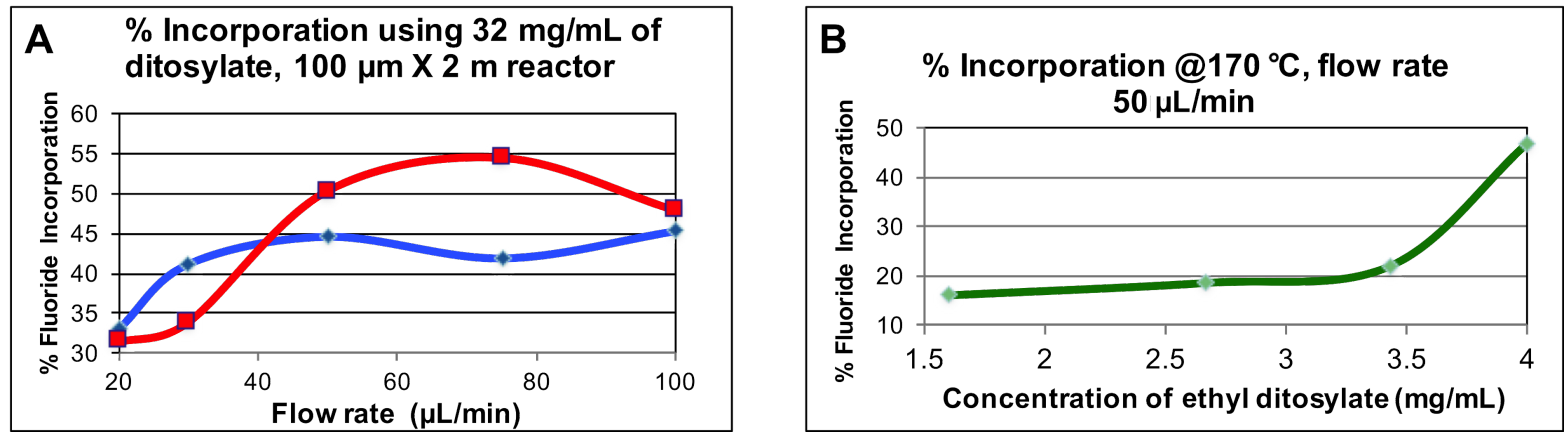
(A) Incorporation yield of [^18^F]fluoride versus flow rate, red line 180 °C, blue line 150 °C, moderately high yields can be obtained; (B) Incorporation yield of [^18^F]fluoride versus ditosylate concentration. An incorporation yield of almost 50% was obtained with only 4 mg/mL.

Using the NanoTek Discovery mode, in the 1-step configuration, the synthesis of [^18^F]fluoroethyltosylate (**10**) was optimized, initially using the reported methods which used up to 32 mg/mL of the ditosylate **9** [17], At this concentration of ditosylate **9**, up to 55% radiochemical conversion (RCC) were noted (Figure 2A). However, if this high concentration is used for the 2-step preparation, it would lead to low product yields due to the competition of the large amounts of unreacted ditosylate with the precursor **8**. By altering the ratios between the [^18^F]fluoride and the ditosylate precursor **9**, the minimum concentration of the ditosylate precursor could be rapidly determined (Figure 2B). The reaction of [^18^F]TEAF with ethylene glycol ditosylate (4 mg/mL, 10 µmol/mL) in DMSO in the first reactor resulted in a final solution concentration of 5 µmol/mL of the ethylene ditosylate, and still yielded ≈45% RCC.

#### Step 2: Reaction of [^18^F]fluoroethyltosylate (**10**) with FEMPT precursor **8**

The [^18^F]fluoroethyltosylate solution, prepared in the first step of the reaction was then mixed with the precursor phenolate **8** (2 mg/mL, 5 µmol/mL) at various flow rates, temperatures and ratios using the Discovery mode of the Advion NanoTek software. The concentration of the precursor was selected such that at a 1:1 ratio, the [^18^F]fluoroethyltosylate and ditosylate solution would not completely consume the precursor during the second step, as there is no purification of the [^18^F]fluoroethyltosylate from the ditosylate prior to the second step. Selected results are shown in Table 2.

**Table 2:** Selected reaction conditions for two-step continuous-flow radiosynthesis.

Flow rate (μL/min)		Reactor temperature (°C)		P2 : Reaction 1 ratio	% Radiochemical yield
P1 and P3 combined	P2	Reactor 1	Reactor 2		

50	70	170	150	2	10
50	100	170	125	1	4
50	100	170	170	1	7
50	50	170	170	1	12
50	50	170	150	1	22

The final product was then purified on a Phenomenex Luna column, 10 × 250 mm, 5 μm, with a mobile phase of 55% MeCN: 45% 10 mM phosphate at a flow rate of 5 mL/min. The HPLC fraction containing the product was collected and diluted with 20 mL of sterile water for injection, then this diluted solution was trapped on a HLB SPE light cartridge, washed with 10 mL of water, eluted from the HLB cartridge with 1 mL ethanol and diluted with 10 mL of 0.9% NaCl solution (saline). [^18^F]FEMPT was obtained in ≈7% isolated radiochemical yield and in >98% radiochemical and chemical purity. The identity of the radiotracer was confirmed by co-injection with the standard (see Supporting Information File 1). The use of microfluidics allowed the optimization of the radiosynthesis in one day. The molar activity of the final product was determined to be >148 GBq/µmol (>4 Ci/µmol) by both UV spectroscopy and mass spectrometry methods and both methods were found to be in agreement. The chemical purity was determined using both UV spectroscopy and mass spectrometry and little chlorinated (<0.1%) and no elimination product was seen by mass spectrometry.

**Figure 3:**
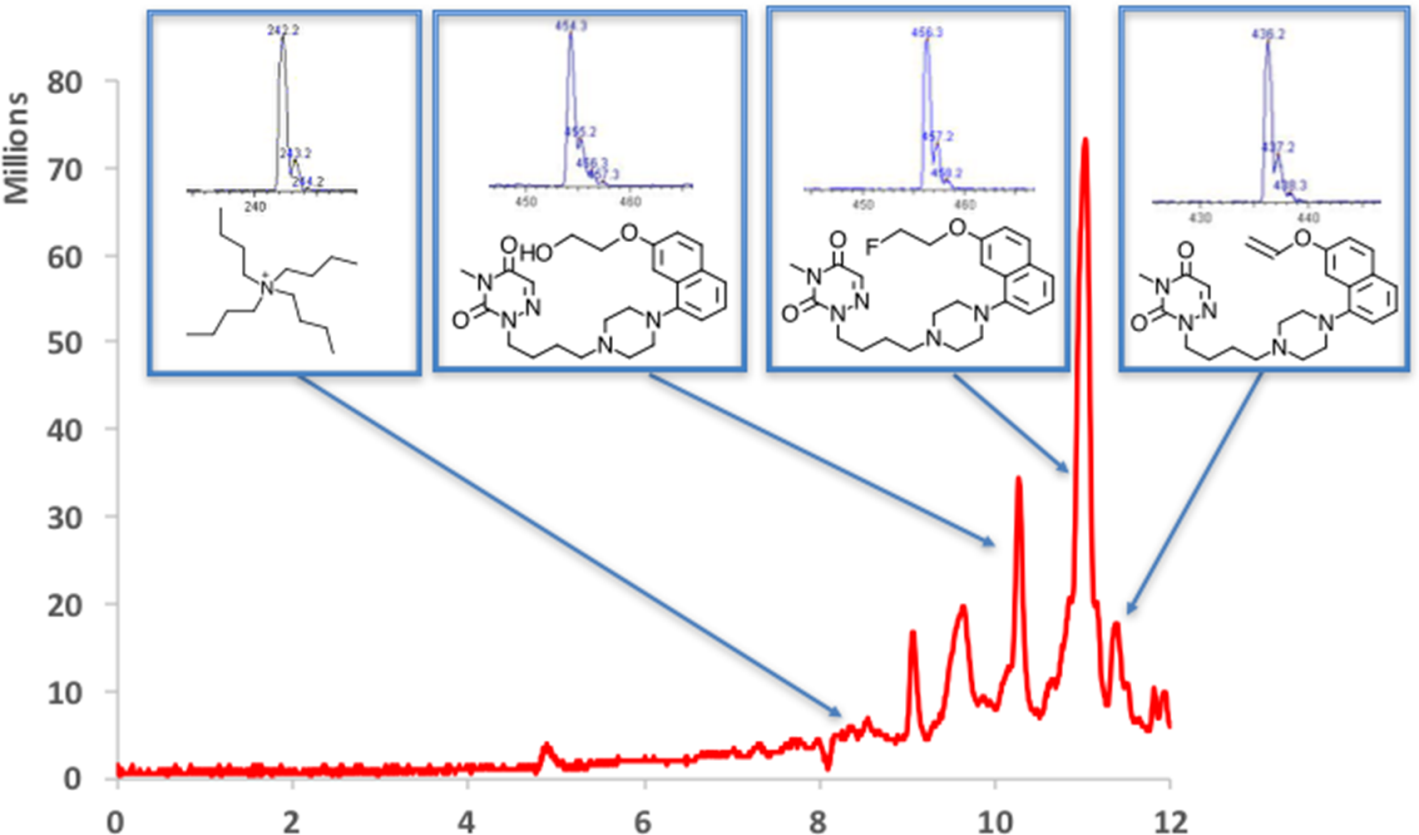
Analysis of the final formulated product by LC–MS, using the trapping system to improve the sensitivity. Red line is the UV spectra observed at 254 nm. Column = 3 µm, 4.6 × 150 mm C18, Phenomenex, Luna. Solvent MeCN:0.1% formic acid, Flow rate = 1 mL/min, Gradient from 20% ACN to 95% ACN at 10 minutes, hold for 2 minutes at 95% MeCN. Insets are the structures identified using the MS data. Mass spectra obtained using an Expression-L Compact Mass Spectrometer (Advion Inc., USA), APCI ion source operating in positive ion mode and corona discharge of 5 μA, *m*/*z* scan range: 200–500.

To determine the products being formed during the radiosynthesis the final formulation was analyzed by LC–MS using 1 mL of the final product solution trapped on a concentration system, which consists of the replacement of the injection loop with a trapping cartridge (Valco Instruments Co. Inc, Houston, TX, Fingertight cartridge assembly #SFECH412, packed with Waters HLB SPE packing material) and the rest of the system remains as standard for the chromatography system. In the UV chromatogram observed on the HPLC system from the direct injection almost no signal is observed. However, when 1 mL of the solution of the purified radiotracer was injected via the trapping system, the UV traces indicate the presence of a number of species with retention times close to the desired product (Figure 3).

A number of the observed impurities were able to be identified using LC–MS and the major impurities observed were the expected elimination product and the hydroxy product. However, all of these materials were below the mass seen for the desired FEMPT and the combined signals were used in the molar activity calculation. Also seen was the presence of tetrabutylammonium salt, which appeared through the semi-preparative purification and the reformulation step. However, the signal for the tetrabutylammonium salt corresponds to <10 µg/mL.

## Conclusion

In summary, [^18^F]FEMPT was efficiently synthesized by continuous flow microfluidics. This protocol is generally applicable for the implementation of a suitable microfluidic process to optimize classical ^18^F-radiofluorination reactions. Preclinical PET imaging studies with this radiotracer are underway.

## Supporting Information

File 1 Experimental part.
