# Supplementary material for: Microfluidic radiosynthesis of [18F]FEMPT, a high affinity PET radiotracer for imaging serotonin receptors
Source: Beilstein J Org Chem. 2017 Dec 29;13:2922–7. doi: 10.3762/bjoc.13.285 (PMC5753126; doi:10.3762/bjoc.13.285)
Supplement: File 1 — Experimental part. [file Beilstein_J_Org_Chem-13-2922-s001.pdf]

Supporting Information

for

Microfluidic radiosynthesis of [^{18}F]FEMPT, a high affinity PET radiotracer for imaging serotonin receptors

Thomas Lee Collier[1,2*], Steven H. Liang[1], J. John Mann[3], Neil Vasdev[2] and J. S. Dileep Kumar[3]

Address: [1]Division of Nuclear Medicine and Molecular Imaging, Massachusetts General Hospital & Harvard Medical School, Boston, MA, USA, [2]Advion, Inc., Ithaca, NY, USA and [3]Molecular Imaging and Neuropathology Division, New York State Psychiatric Institute, New York, NY, USA

Email: Thomas Lee Collier - tcollier@mgh.harvard.edu

* Corresponding author

Experimental part

Synthesis of 2-(4-(4-(7-(2-fluoroethoxy)naphthalen-1-yl)piperazin-1-yl)butyl)-4-methyl-1,2,4-triazine-3,5(2*H*,4*H*)-dione (**7**)

1-Bromo-2-fluoroethane (26.4 mg, 0.208 mmol) and potassium carbonate (96 mg, 0.694 mmol) were added to the solution of 2-(4-(4-(6-hydroxypyridin-2-yl)piperazin-1-yl)butyl)-4-methyl-1,2,4-triazine-3,5(2*H*,4*H*)dione (**6**, 50 mg, 0.139 mmol) in DMF (2 mL). The reaction mixture was heated at 80 °C for 4 h. The reaction mixture was cooled to rt, diluted with water, extracted with ethyl acetate (3 × 30 mL). The combined organic layers were washed with brine, dried over $MgSO_4$ and concentrated under vacuum. The crude residue was column chromatographed using 2% methanol in CH_2Cl_2 to give the compound **5** as a viscous liquid (38 mg, 70%).

^1H NMR (400 MHz, $CDCl_3$) δ 7.9 (d, 1H), 7.6 (m, 2H); 7.5 (s, 1H), 7.3 (m, 2H), 7.2 (dd, 1H), 7.1 (d, 1H), 4.87 – 4.80 (m, 1H), 4.75 – 4.67 (m, 1H), 4.34 – 4.26 (m, 1H), 4.25 – 4.16 (m, 1H), 4.02 (t, 2H), 3. 4 (s, 3H), 3.2 (brs, 4H), 2.6 (brs, 4H), 2.4 (t, 2H), 1.86 – 1.74 (m, 2H), 1.59 (m, 2H); HRMS calcd for $C_{24}H_{30}FO_3N_5$ (MH+): 456.2354; Found: 456.2268.

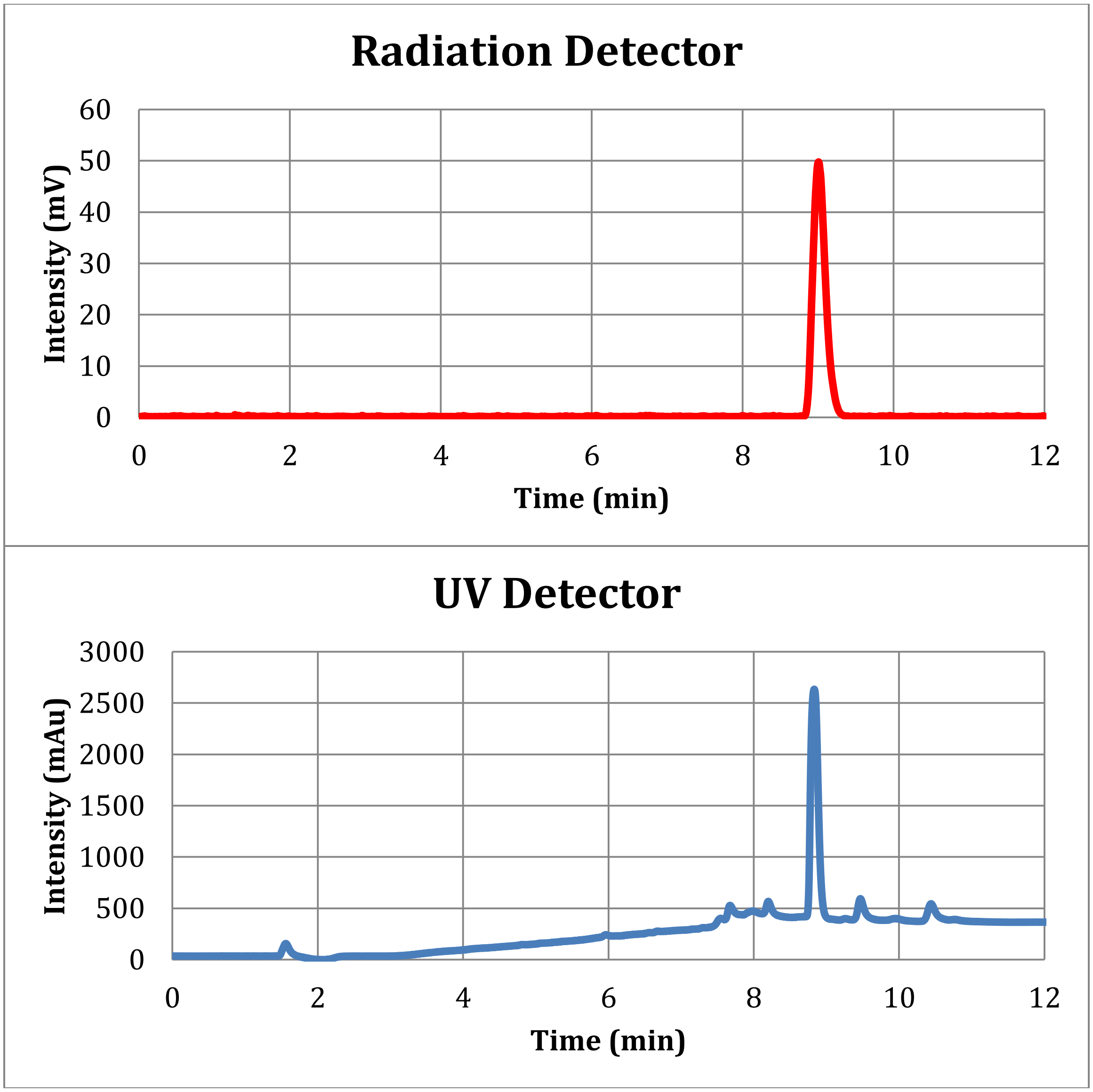

Column : 3 μm, 4.6 × 150 mm C18, Phenomenex, Luna.
Flow rate : 1mL/min,
Solvent : Acetonitrile : 0.1% formic acid,
Gradient: 0–1 min : 10% Acetonitrile : 0.1% formic acid
1–7 min : gradient to 95% Acetonitrile : 0.1% formic acid
7–11 min : 95% Acetonitrile : 0.1% formic acid
11–12 min : gradient to 10% Acetonitrile : 0.1% formic acid
